# A Novel Satellite PRN Code Assignment Method Based on Improved RLF Algorithm

**DOI:** 10.3390/s22155538

**Published:** 2022-07-25

**Authors:** Weiwei Wang, Ye Tian, Lang Bian, Guoyong Wang, Yansong Meng, Lixin Zhang

**Affiliations:** China Academy of Space Technology (Xi’an), Xi’an 710100, China; wangweiwei_504@163.com (W.W.); tianye_504@163.com (Y.T.); bianl35@163.com (L.B.); wangguoyong321@163.com (G.W.); mengys_504@163.com (Y.M.)

**Keywords:** LEO-based navigation augmentation, navigation signal receiver, PRN code, average acquisition time, RLF algorithm

## Abstract

Low Earth Orbit (LEO) satellites have stronger received signals and more rapid geometry changes than Global Navigation Satellite System (GNSS) satellites, making them attractive for positioning, navigation, and timing (PNT) applications. Due to the low altitude, the LEO constellation requires more satellites to cover the entire globe and more Pseudo Random Noise (PRN) codes to realize Code Division Multiple Access (CDMA), which means greater receiver storage resources and receiver acquisition time. In this paper, different from the traditional methods that assign a unique PRN code to each satellite, we propose a novel method in which several satellites share the same PRN code, and simply demonstrate the feasibility and benefits of this method. To determine the minimum number of PRN codes needed for a constellation, we build a mathematical model. After the algorithm comparison, we improve the recursive largest first (RLF) algorithm so that it has a higher running speed and a smaller approximate optimal solution within a certain time period. By studying polar-orbiting and walker constellations, we find that if other satellite parameters remain the same, the higher the orbital altitude is, the more PRN codes are needed, and no matter what the orbital inclination is, the minimum number of PRN codes remains the same. Overall, it is feasible and meaningful for several satellites sharing the same PRN code to save storage resources and reduce the satellite acquisition time of the receiver. If this new technology is applied, the storage resources and the average satellite acquisition time of the receiver will be, at most, one-third of previous ones.

## 1. Introduction

Nowadays, the GNSS can stably provide the location, time, and speed information for global users and plays a huge role in geoscience and engineering applications [1]. However, with the development of new technologies, such as 5G/6G, the Internet of Things (IoT), artificial intelligence, and autonomous driving, users’ demand for accurate space-time information is changing [2]. For example, the intelligent transportation represented by autonomous driving requires a fast-converging and high-precision navigation service. The navigation service provided by the current GNSS has limitations, so it may be unsuitable for new technologies in the future. First of all, the positional accuracy of GNSS basic navigation service is about 10 m, which does not meet the needs of high-precision positioning. Secondly, GNSS signals have large signal spatial attenuation and cannot provide reliable and continuous navigation in areas, such as overpasses, indoor areas, forests, and urban canyons. In addition, because of the low signal power, GNSS signals are vulnerable to jamming and spoofing [3]. Determining how to improve the service performance of satellite navigation systems is a key issue in the field of navigation.

The LEO-based navigation augmentation system has been shown to complement the current GNSS service effectively and augment its positioning and navigation performance [4,5], so it has become a research hotspot in the satellite navigation field. Generally, there are two types of LEO-based navigation augmentation system: information augmentation and signal augmentation. The use of the LEO constellation in signal augmentation systems has so many advantages by which it can improve the service performance of the GNSS. First of all, the LEO satellites undergo rapid geometry changes [5], which is beneficial for the rapid convergence of PPP (precise point positioning). The results of Ge et al. [6] showed that LEO-augmented GNSS (GPS + BDS + Galileo) can achieve centimeter-level accuracy within 5 min of PPP convergence. Li et al. [7] discovered that having more LEO satellites leads to a shorter convergence time, and the convergence time can be shortened from 8.2 to 0.8 min by introducing observations from 288 polar-orbiting LEO satellites. The results of Wang et al. [8,9] showed that the LEO augmentation could be significantly reduced the convergence time of the ambiguity-float formal horizontal precision. Secondly, LEO signal augmentation can provide new observations. For occluded environments, such as under overpasses, forests, urban canyons, and even indoors, positioning can be compensated by stronger signals broadcast by the LEO. Taking Iridium as an example, Satellite Time and Location (STL) is a PNT service that was made available through Iridium by Satelles in 2016 [10]. Satelles company tested the STL’s service performance indoors in a high-rise building and found an indoor positioning accuracy of 20 m and a timing accuracy at the sub-microsecond level [11,12]. In addition, due to the lower orbit, navigation signals from the LEO satellite are stronger, which adds robustness to the navigation service [13]. For example, Iridium signals are 300 to 2400 times stronger than GNSS on the ground [14], and users have already employed LEO-based signals in environments with high GNSS interference [15]. It is worth recalling that OneWeb [16], SpaceX [17], Boeing [18], China Aerospace Science, Technology (CAST) Corporation [19], and other internationally renowned companies have worked out their plans for LEO constellations with hundreds or thousands of satellites, and Reid et al. successfully demonstrated the potential of these LEO constellations to be leveraged as a platform to provide navigation services [20].

In the future, in order to achieve navigation enhancement, LEO constellations such as Weili, Hongyan and Luojia will broadcast traditional CDMA signals, which means that each LEO satellite has a unique PRN code. However, for hundreds or thousands of satellites from the LEO signal augmentation system, the traditional method places a huge burden on the navigation receiver and PRN code design, while the traditional method is suitable for GNSS constellations consisting of dozens of satellites. Specifically, signal augmentation means that LEO satellites transmit navigation signals independently. Similar to GNSS signals, the LEO navigation signal structure consists of three layers: the carrier wave, PRN code (C/A code for civil and P-code for military), and navigation data. Because LEO satellites are located 500–1500 km away [20], the LEO constellation requires dozens to thousands of satellites to cover the globe (shown in Table 1) and the same number of PRN codes to realize CDMA when using the traditional method where each LEO satellite in a constellation has a unique PRN code. On the one hand, it is difficult to design thousands of PRN codes with good autocorrelation properties and high cross correlation properties. This design requires the different PRN codes for each LEO satellite to be nearly orthogonal, which is very difficult for 1023-chip C/A-code to design hundreds or thousands of PRN codes. On the other hand, if there are thousands of PRN codes, the navigation signal receiver needs more time to search and more storage resources to store PRN codes during satellite acquisition, which is not feasible for small and medium-sized receivers. If the number of PRN codes can be reduced, the burden on the receiver and PRN code design may be greatly reduced.

However, at present, scholars’ research focuses on modulation methods in the LEO navigation signal field. The phase-coded linear chirp signal is designed by Harshal AR [28], which is immune to Doppler frequency offset and reduces multiple access interference. Li et al. [29] design a chip level pulse quasicontinuous navigation signal, which is compatible with GNSS. Xue et al. [30] propose A universal modulation scheme based on the continuous phase modulation (CPM) family. There is little research on PRN design or assignment of LEO navigation signals.

In order to reduce the number of PRN codes and burden on the navigation receiver, we propose a new method where several satellites share the same PRN code. Obviously, the new method can bring benefits to the navigation signal receiver and PRN code design. However, it may make it difficult for users to distinguish the satellite that the signal is being broadcast from. If the same user cannot simultaneously observe signals from satellites sharing the same PRN code at any time, each PRN code will be used, at most, once within the user’s visual range, and users will be easily able to distinguish satellite signals. Therefore, we need to design a grouping scheme for satellites so that the same user on the ground cannot simultaneously observe the same PRN code at any time.

As shown in previous studies, each LEO satellite in a constellation has a unique PRN code in a LEO-based navigation signal augmentation system. In this study, for the first time, we group the LEO satellites so that several satellites can share the same PRN code. The benefits for the acquisition of receiver are analyzed. This paper is organized as follows: In Section 2, we demonstrate the feasibility and benefits of this method and provide the mathematical model and calculation process used for the minimum shared PRN codes. To evaluate the performance of six common algorithms for this problem, we design 6 LEO constellations with different satellite numbers, orbital altitudes, and types of orbits, and group satellites using these six algorithms. We show that the RLF algorithm performs best, and this is introduced and improved in Section 3. Afterwards, in Section 4, we take the polar-orbiting, walker, and hybrid constellations as experimental objects and study the effect of satellite orbital parameters on the number of minimum PRN codes. Finally, the discussion and conclusions are provided in Section 5 and Section 6.

## 2. Methods and Mathematical Models

This section begins with the feasibility and benefits of this method for signal acquisition. Then, the mathematical model is established for the minimum shared PRN codes, which is similar to the optimum graph coloring problem. Finally, we describe the calculation process of LEO satellite PRN code assignment method.

### 2.1. Feasibility and Benefits

**Figure 1:**
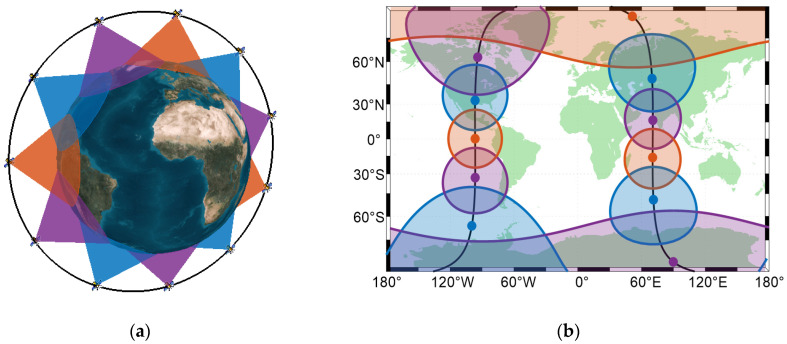
Satellite PRN code assignment diagram of a constellation composed of 11 satellites. Three colors represent three PRN codes, which are enough to realize CDMA for 11 satellites. (**a**) Three-dimensional view of the PRN code assignment; (**b**) Two-dimensional view of the PRN code assignment. The points are satellite positions. The circles are the range in which the satellite PRN codes can be received on the ground.

We believe that it is feasible and meaningful for several satellites to share the same PRN code to save the storage resources of the receiver. As shown in Figure 1, 11 satellites are evenly distributed in a polar-orbiting orbit. With the traditional method, 11 PRN codes are required for 11 satellites to broadcast navigation signals. If 3–4 satellites share one PRN code, 3 PRN codes are enough. In the case of Figure 1, two satellites with the same PRN code cannot be seen at all locations on Earth at the same time. If this new technology is applied, the storage resources of the receiver will be three-elevenths the size of the previous one.

**Figure 2:**
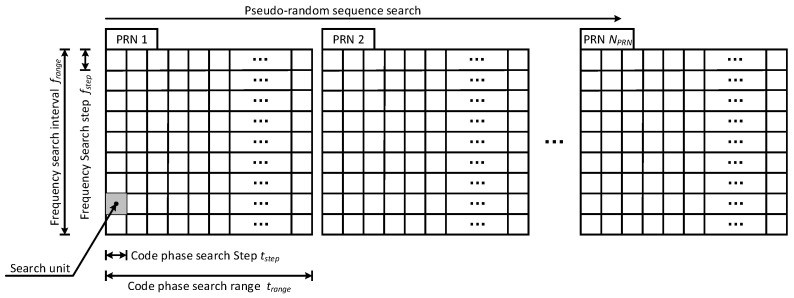
The search process for navigation signal acquisition.

We also believe that using several satellites to share the same PRN code is useful to shorten the satellite acquisition time of the receiver. The basic idea of acquisition is to despread the navigation signal and determine the carrier frequency, code phase, and PRN code that match the signal. If the correct PRN code with the correct phase is multiplied on the navigation signal, the signal will become a continuous waveform signal. Figure 2 shows the process of navigation signal acquisition. Navigation signal acquisition consists of a three-dimensional search to synchronize a receiver-generated reference signal with the received navigation signal.

In terms of the frequency, the frequency search range is $f_{range}$, and the step is $f_{step}$. In terms of the code phase, the code phase search range is $t_{range}$, and the step is $t_{step}$. The PRN code search range is $1\sim N_{PRN}$. There are $t_{range}f_{range}/t_{step}f_{step}$ search units. The dwell time $T_{dwell}$ is the time required for the receiver to perform one signal search in each search unit, so the time used to search the whole indefinite interval is

(1) $$T_{tot}=\frac{t_{range}f_{range}}{t_{step}f_{step}}\cdot N_{PRN}\cdot T_{dwell}$$

If a signal is detected during the search process, the search should stop. Assuming that the parameters are uniformly randomly distributed, the average acquisition time is estimated as

(2) $$T_{avg}=\frac{1}{2}\cdot \frac{t_{range}f_{range}}{t_{step}f_{step}}\cdot N_{PRN}\cdot T_{dwell}$$

From the above formula, it can be seen that the average acquisition time is associated with the number of PRN codes, and the more PRN codes there are in the navigation system, the greater the average time of acquisition is. Therefore, to reduce the average acquisition time of navigation receivers, we should assign as few PRN codes to the LEO constellation as possible.

### 2.2. Mathematical Model

The problem with optimum satellite PRN code assignment is similar to the optimum graph coloring problem. The graph coloring problem can be depicted as follows: Given a graph $G=\left(V,E\right)$ with vertex set V and edge set E, the process of coloring the vertex set V with a set $C=\left\{1,2,\ldots ,k\right\}$ containing at least k colors can be described as a mapping f:V→C, and for any two different vertices $v_{i},v_{j}\in V$, if there is an edge $\left(v_{i},v_{j}\right)\in E$, then $f\left(v_{i}\right)\neq f\left(v_{j}\right)$. The minimum number of colors, which is denoted by $\chi \left(G\right)$, is called the chromatic number of the graph.

**Figure 3:**
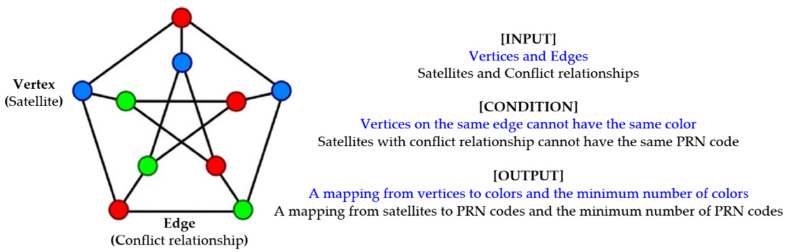
The consistency description of the two problems from the perspective of input, condition and output. The graph on the right is a model of graph coloring problem. The description of the graph coloring problem is in blue, and the description of the satellite PRN code assignment problem is in black.

We consider the satellites to be vertices that form the set V. We consider both satellites $v_{i},v_{j}\in V$ with conflict as edges and form the set E. We treat the PRN code set $C=\left\{1,2,\ldots ,k\right\}$ as a color set. The satellite PRN code assignment problem can be depicted as follows: Given a graph $G=\left(V,E\right)$ with satellite set V and conflict relationship set E, the process of assigning PRN codes to satellite set V with a set $C=\left\{1,2,\ldots ,k\right\}$ containing at least k PRN codes can be described as a mapping f:V→C, and both satellites with conflict $\left(v_{i},v_{j}\right)\in E$ cannot have the same PRN code $f\left(v_{i}\right)\neq f\left(v_{j}\right)$. The minimum number of PRN codes, which is denoted by $\chi \left(G\right)$, is needed. Figure 3 descript the consistency of the two problems in terms of input, condition and output. Therefore, the models of the satellite PRN code assignment problem and graph coloring problem are consistent, and the algorithms available for graph coloring can also be applied to this problem.

According to the above definition, the mathematical model of the satellite PRN code assignment problem can be further summarized. It is assumed that there are $N_{sat}$ satellites, $v_{1},v_{2},\ldots ,v_{N_{sat}}$, where there are $N_{PRN}$ optional PRN codes for each satellite and the set is $C_{i}=\left\{1,2,\ldots ,N_{PRN}\right\}$. The mathematical model of the satellite PRN code assignment problem is as follows:

(3) $$\begin{array}{c} \min\chi \left(G\right)=\sum_{k=1}^{N_{PRN}}\delta \left(\sum_{i=1}^{N_{sat}}x_{ik}\right)\\ s.t.\left\{\begin{array}{l} x_{ik}\in \left\{0,1\right\},\;\;\;\;\;\;\;\;\;\;\;\;\;\;\;\;\;\;\;\;\;\;\;\;\;\;\;\;\;\;\;\;1\leq i\leq N_{sat},\;\;\;\;1\leq k\leq N_{PRN}\\ \sum_{k\in C}x_{ik}=1,\;\;\;\;\;\;\;\;\;\;\;\;\;\;\;\;\;\;\;\;\;\;\;\;\;1\leq i\leq N_{sat},\;\;\;1\leq k\leq N_{PRN}\\ \sum_{\left(v_{i},v_{j}\right)\in E}x_{ik}\cdot x_{jk}=0,\;\;\;\;\;\;\;\;\;\;1\leq i,j\leq N_{sat},\;\;\;1\leq k\leq N_{PRN} \end{array}\right. \end{array}$$

where the function $\delta \left(z\right)$ is a 0–1 function,

(4) $$\delta \left(z\right)=\left\{\begin{array}{l} 1,\;\;\;\;\;\;\;\left(z>0\right)\;\;\;\\ 0,\;\;\;\;\;\mathrm{otherwise} \end{array}\right.$$

and for the variable $x_{ik}$,

(5) $$x_{ik}=\left\{\begin{array}{l} 1,\;\;\;\;\;\;\mathrm{The}\;\mathrm{PRN}\;\mathrm{code}\;\mathrm{assigned}\;\mathrm{to}\;\mathrm{satellite}\;v_{i}\;\mathrm{is}\;k\;\;\;\;\;\;\;\\ 0,\;\;\;\;\;\;\mathrm{The}\;\mathrm{PRN}\;\mathrm{code}\;\mathrm{assigned}\;\mathrm{to}\;\mathrm{satellite}\;v_{i}\;\mathrm{is}\;\mathrm{not}\;k \end{array}\right.$$

### 2.3. Calculation Process

The calculation of the minimum number of PRN codes can be divided into four steps. The details are as follows: 

Firstly, we set the users’ positions and simulated the satellite orbits. A total of 65,160 ground stations were established at intervals of 1 degree latitude and 1 degree longitude on the ground. These were regarded as user positions, and the user elevation threshold $e_{\mathrm{threshold}}$ was set as 7 degrees. By simulating the satellite orbit for a long time, the elevation e from the user to the satellite at each moment was obtained through the position of the satellite and user according to the following formula.

(6) $$e=\arctan\left(\frac{\cos\left(\phi_{1}-\phi_{2}\right)\times \cos\theta_{1}-0.15127}{\sqrt{1-{\left[\cos\left(\phi_{1}-\phi_{2}\right)\times \cos\theta_{1}\right]}^{2}}}\right)=\left\{\begin{array}{c} >e_{\mathrm{threshold}},\;\;\;\;\;\;\mathrm{can}\;\mathrm{be}\;\mathrm{received}\;\;\;\;\\ <e_{\mathrm{threshold}},\;\;\;\mathrm{can}\;\mathrm{not}\;\mathrm{be}\;\mathrm{received} \end{array}\right.$$

where $\phi_{1}$ and $\theta_{1}$ are the longitude and latitude of the user; $\phi_{2}$ is the longitude of the satellite. If the elevation angle from the user to the satellite was higher than the user elevation threshold, we deemed that the satellite navigation signals could be received by users.

Secondly, we obtained the satellite conflict relationships based on the positions of users and satellites. The PRN code transmitted by the satellite has the CDMA function. Therefore, when sharing PRN codes, it is essential to ensure that the satellites broadcasting the same PRN code cannot appear in the user’s view at any position on the Earth. We defined the conflict relationship of a satellite as follows: a signal of satellites can be received by the same user at a certain time, and the satellites with a conflict relationship cannot share the same PRN code. If a constellation consists of $N_{sat}$ satellites, a matrix R of size $N_{sat}\times N_{sat}$ is established to record the conflict relationship between satellites, and the matrix R is defined as follows:

(7) $$R\left(i,j\right)=\left\{\begin{array}{l} 1,\;\;\;\mathrm{signal}\;\mathrm{of}\;\mathrm{Satellite}\;i\;\mathrm{and}\;j\;\mathrm{can}\;\mathrm{be}\;\mathrm{received}\;\mathrm{by}\;\mathrm{the}\;\mathrm{same}\;\mathrm{user}\;\mathrm{at}\;a\;\mathrm{certain}\;\mathrm{time}\;\;\;\;\;\;\;\;\;\;\;\;\;\;\;\;\;\\ 0,\;\;\;\mathrm{signal}\;\mathrm{of}\;\mathrm{Satellite}\;i\;\mathrm{and}\;j\;\mathrm{cannot}\;\mathrm{be}\;\mathrm{observed}\;\mathrm{by}\;\mathrm{the}\;\mathrm{same}\;\mathrm{user}\;\mathrm{at}\;\mathrm{any}\;\mathrm{time}\;\mathrm{or}\;\left(i=j\right) \end{array}\right.$$

We simulated the satellite orbit for as much time as possible. If the matrix R did not change within a certain time (several period of satellite), we considered that all satellite conflicting relationships had been collected.

Thirdly, we built the computational model, which is similar to the optimum graph coloring problem. The graph coloring problem has been proven to be a NP complete problem. There is currently no algorithm to find the optimal coloring in a time bounded by a polynomial in the graph [31]. Fortunately, there are many algorithms to solve the problem sub-optimally and obtain the approximate optimum solution, from which the most suitable algorithm for the optimum satellite PRN code assignment problem could be found. According to the characteristic that the LEO satellites are usually uniformly distributed, we improved the algorithm to make it more suitable for our question.

Finally, we obtained an approximate optimal solution with a suitable algorithm and verified its feasibility. Meanwhile, a satellite PRN code assignment scheme, corresponding to the approximate optimal solution could be obtained, and we brought the scheme back to the satellite constellation in order to verify whether this satellite PRN code assignment scheme was feasible.

## 3. Algorithm

In this section, we design 6 LEO constellations to evaluate the performance of 6 common algorithms for this problem. Subsequently, according to the characteristic that the LEO satellites are usually uniformly distributed, we improve the RLF algorithm to make it more suitable for our question.

### 3.1. Algorithm Comparison

The above analysis shows that the problem of optimum satellite PRN code assignment is similar to the minimum graph coloring problem, so we studied the algorithms that solve the graph coloring problem and tried to apply these algorithms to calculate the minimum number of PRN codes. The minimum graph coloring problem has been shown to be an NP complete problem, and there is currently no algorithm to find the optimal solution in a time bounded by a polynomial in the graph. Fortunately, there are many algorithms that can solve the problem sub-optimally to obtain the approximate optimum solution, e.g., the depth-first search (DFS) [32], genetic algorithm (GA) [33], greedy algorithm [34], ant colony optimization (ACO) [35], simulated annealing (SA) [36], RLF [37], and so on. In order to find the most suitable algorithm for the optimum satellite PRN code assignment problem, we selected 6 algorithms and used these to calculate the minimum number of PRN codes. These 6 algorithms are described briefly.

DFS obtains the minimum number of PRN codes by traversing all possibilities, and the minimum value can be obtained in theory. However, the time complexity of DFS is exponential, which means it is difficult to reach the optimal solution within a limited time. 

Both GA and SA generate a random PRN code assignment scheme. If the assignment scheme error occurs, it will be included in the cost: the more errors that occur, the greater the cost is. The assignment scheme is changed according to a certain probability of mutation/disturbance, meaning that the cost might be decrease. However, the convergent solution may not be the approximate optimal solution, so multiple calculations are required.

The greedy algorithm can be described as follows: satellites are arranged in a random order, as many satellites as possible are selected to share the same PRN code in order, and if the remaining satellites cannot share this PRN code, the next PRN code is assigned. The greedy algorithm can be calculated within a short time, but the calculation result depends on the order of the satellites, and the approximate optimal solution can be obtained through repeated calculations by constantly disrupting the order of the satellites.

ACO and RLF can be regarded as improved greedy algorithms. In ACO, a group of ants is released, and results are obtained by pheromone and greedy methods. Then, the pheromone is added to the correct path, and the ants are released again and again, until an approximate optimal solution is obtained. On the basis of greed, the RLF algorithm preferentially selects large-degree satellites so that more satellites may be assigned to the same PRN code.

**Figure 4:**
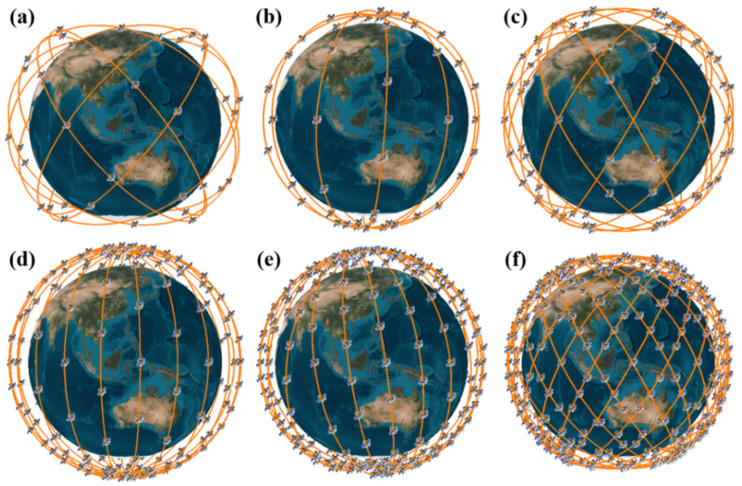
Designed LEO constellations: (**a**) 48 LEO, 1414 km, walker; (**b**) 66 LEO, 778 km, polar; (**c**) 120 LEO, 1000 km, walker; (**d**) 192 LEO, 1200 km, polar; (**e**) 288 LEO, 1000 km, polar; (**f**) 576 LEO, 900 km, walker.

To evaluate the performance levels of these 6 common algorithms in relation to this problem, we designed 6 LEO constellations with different satellite numbers, orbital altitudes, and types of orbits using simulation software and then obtained the minimum number of PRN codes using these 6 algorithms. Figure 4 shows the designed LEO constellations and two typical orbits, including the polar-orbiting orbit and the walker orbit. In particular, the LEO-48 constellation is borrowed from the Globalstar constellation [21], and the LEO-66 constellation scheme is related to the Iridium constellation [38].

Table 2 shows the detailed orbital configurations and approximate minimum solutions of the designed LEO constellations. The ratios in the table represent the satellite number compared with the minimum number of PRN codes. The results of this ratio further demonstrate its benefits for the navigation signal receiver, and this method can greatly reduce the storage resources needed and the average satellite acquisition time of the receiver.

**Figure 5:**
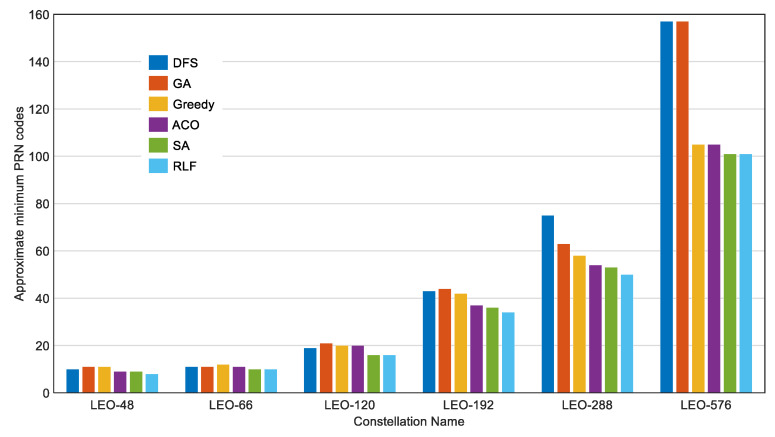
Comparison of the optimal approximate solutions for the 6 algorithms. The parameters and methods used in the algorithm are as follows: **DFS**, We assigned PRN codes to satellites with few optional PRN codes as soon as possible; **GA**, The number of populations was 300, the Max Generation was 2000, and the program needed to be run multiple times; **Greedy**, The number of iterations was 100,000; **ACO**, 50 ants walked 2000 times, the decay factor was 0.99, and the program needed to be run multiple times; **SA**, The initial temperature was 10^6^, the temperature decay factor was 0.9, and the program needed to be run multiple times; **RLF**: The number of iterations was 20,000.

Figure 5 shows a comparison of the optimal approximate solutions for the 6 algorithms. The height of the column in the figure is the approximate minimum number of PRN codes for the 6 constellations calculated by the 6 algorithms. DFS and GA are shown to be less effective when the number of satellites is large. The RLF algorithm and SA have better results on all 6 constellations, and the RLF algorithm is slightly more suitable for calculating the approximate minimum number of PRN codes. We chose the RLF algorithm for further analysis, and this is introduced and improved in the next section.

### 3.2. Algorithm Improvement

In the previous subsection, we showed that compared with the other five algorithms, the RLF algorithm is the most suitable for calculating the minimum number of PRN codes. The RLF algorithm was originally proposed by Frank Leighton in 1979, in part for use in constructing solutions to large timetabling problems [39]. Paper [40] proposed a variant of RLF, which is called LAZY RLF, that exploits possible savings in the computations during algorithm execution. In spite of its best results in terms of quality, the RLF heuristic has been seldom used in the literature [40]. 

Because the satellites are usually uniformly distributed in the sky and the constellation is symmetrical, the conflicts of satellites have certain characteristics. We used these characteristics to improve the RLF algorithm. Before presenting the improvement, we introduce the RLF algorithm process, and the specific steps used in the RLF algorithm are as follows:

- Step 1: Initialization. Set q to 0, and set U=V. q indicates the current PRN code and U represents the set of unnumbered satellites. Proceed to 2.

(8) q:=0;  U∶=V

- Step 2: Using the new PRN code, increase q by 1 and set $C_{q}$ to empty. Then, proceed to 3.

(9) $$q:=q+1;\;\;\;C_{q}:=\phi$$

- Step 3: Find the satellite set W, and number each satellite in set W as q. There are two conditions. The first is that all satellites $v_{i}\;\in W$ must be unnumbered, and the second is that all satellites $v_{i}\;\in W$ must not conflict with satellites in $C_{q}$. Proceed to 4.

(10) $$\begin{array}{c} W=\left\{v_{i}\right\},\;\;\;\;v_{i}\in U\\ R\left(i,j\right)=0,\;\;\;\;v_{i}\;\in W,\;\;\;v_{j}\in C_{q} \end{array}$$

- Step 4: Compute the degree of all satellites in W and get the maximum degree. The satellite degree is the number of distinct PRN codes assigned to other non-conflicting satellites. Move all satellites with the maximum degree to $W_{0}$. Proceed to 5.

(11) $$\begin{array}{c} W_{0}=\left\{v_{j}\right\},\;v_{j}\in \;W\\ deg(v_{j})=\max deg(v_{i}),\;\;v_{i}\;\in W,\;\;\;v_{j}\;\in W_{0} \end{array}$$

- Step 5: Choose a satellite $v_{k}\in W_{0}$ at random and assign a PRN code q to satellite $v_{k}$. In other words, move $v_{k}$ to $C_{q}$ and remove $v_{k}$ from U. Return to Step 3.

(12) $$v_{k}\in C_{q}\;\;v_{k}\notin U$$

- Step 6: Check whether U=ϕ. If so, all satellites are numbered, and the program should be stopped. If not, no other satellite fits this PRN code q, so return to Step 2.

In this study, we improved the RLF algorithm and evaluated the performance of the algorithm before and after the improvement. Satellites of a polar-orbiting or walker configuration, which are commonly used in the LEO constellation, are usually equally distributed at the same orbital altitude. Therefore, in Step 4 of the RLF algorithm, when computing the degree of all satellites in W, we found that, in some cases, many satellites’ degrees are equal to the maximum degree, and there are many satellites in the set $W_{0}$. In step 5, a satellite in set $W_{0}$, which may consist of many satellites, is randomly selected. The satellite selected has a great influence on the calculation result. To improve the performance of the algorithm, we optimized the way in which the random selection in step 5 is conducted.

We combined all satellites in pairs to get $N_{sat}\cdot \left(N_{sat}-1\right)/2$ satellite pairs. We composed a set of satellites that conflict with satellite $v_{i}$ and a set of satellites that conflict with satellite $v_{j}$ and calculated the union of two sets to obtain the conflict set of satellite pairs $\left(i,j\right)$ consisting of satellites $v_{i}$ and $v_{j}$. When the number of elements in this union is minimal, we believe that this satellite pair can share the same PRN code. These satellite pairs were recorded, and a set P of satellite pairs was formed. In step 5, we preferentially selected the satellite pairs in set P. We replaced step 5 with the following:

- Step 5: Choose a satellite $v_{k}\in W_{0}$ at random and assign PRN code q to satellite $v_{k}$. In other words, move $v_{k}$ to $C_{q}$ and remove $v_{k}$ from U. Return to Step 3.

(13) $$\begin{array}{c} If\;exist\;\left(i,j\right)\in P\;\&\&\;\;v_{i},v_{j}\in W_{0}\;\;\;then\;\;\;v_{i},v_{j}\in C_{q}\;\;\;\;\;\;v_{i},v_{j}\notin U\\ Otherwise\;\;randomly\;choose\;v_{k}\in W_{0}\;\;\;then\;\;\;v_{k}\in C_{q}\;\;v_{k}\notin U \end{array}$$

**Figure 6:**
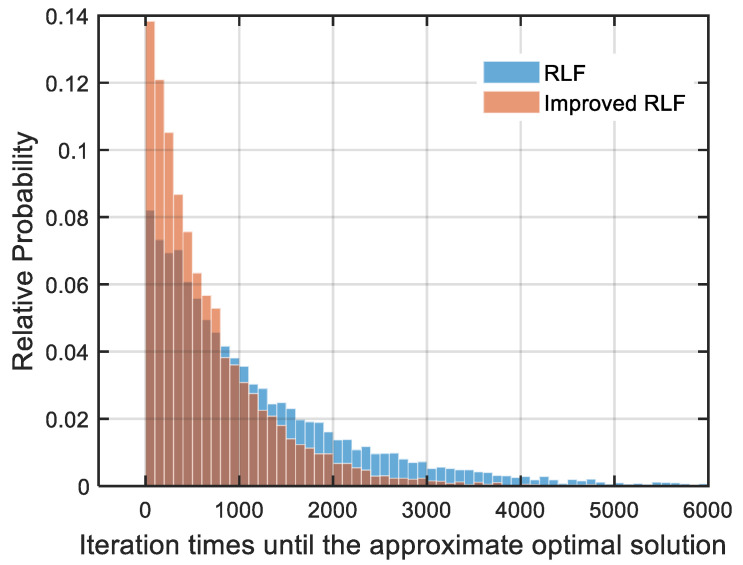
Comparison of the calculation speed between the RLF algorithm and the improved-RLF algorithm. The satellite configuration was the walker 120/12/6 at an altitude of 1000 km. Both RLF and improved-RLF were run 10,000 times, and the number of iterations was 6000. With a probability of 0.0119, the solution of RLF algorithm was found to be greater than the approximate optimal solution, while in all experiments, the optimal solution was obtained in 6000 iterations with the improved-RLF algorithm.

In order to evaluate the performance of the algorithm before and after the improvement, we used LEO-120 as the study object, and the approximate minimum PRN code of the LEO-120 is 16. Due to the randomness of the RLF algorithm, we needed to iterate many times to get the approximate optimal solution. We repeated the experiment 10,000 times and recorded the number of iterations required for the RLF and the improved-RLF to obtain the approximate optimal solution in each experiment. Obviously, the more iterations there is, the greater the computational time of the algorithm is. A histogram of the number of iterations is shown in Figure 6, and the average number of iterations for the RLF is 1138.411, while the average number of iterations for the improved-RLF is 668.752. As can be seen from the figure, in most of the experiments, the RLF algorithm reached the optimal solution within 5000 iterations, while the improved-RLF algorithm reached the optimal solution within 3000 iterations. Overall, through the experiments, we obtained the conclusion that the calculation time for the improved RLF algorithm is faster than that of the RLF algorithm for the problem of calculating the minimum number of PRN codes. 

**Figure 7:**
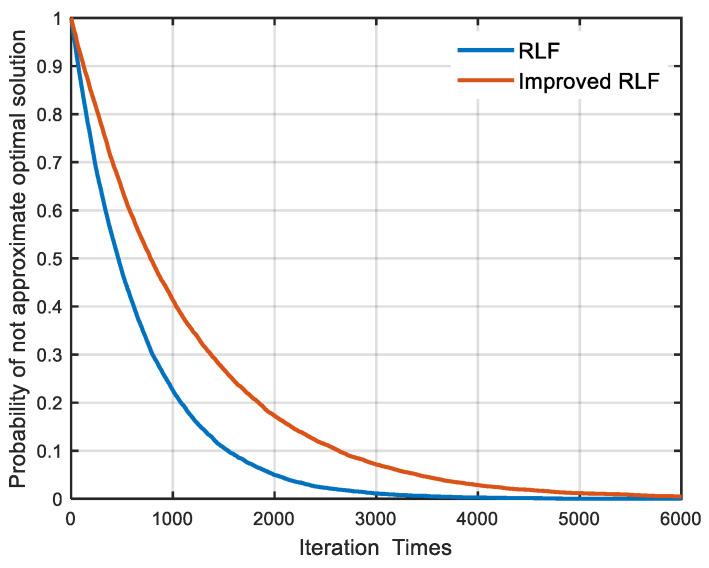
The probability of not approximate optimal solution varying with the number of iterations. The experimental method is the same as that in Figure 6.

Due to the randomness of the RLF algorithm, the convergent solution of RLF algorithm may be not an approximate optimal solution. In order to evaluate the uncertainty, we analyzed the uncertainty based on the above experimental data, as shown in Figure 7. The probability of a non-approximate optimal solution of the RLF algorithm is greater than that of an improved-RLF algorithm. In order to obtain the approximate optimal solution, the number of iterations should be increased as much as possible during the calculation process.

**Figure 8:**
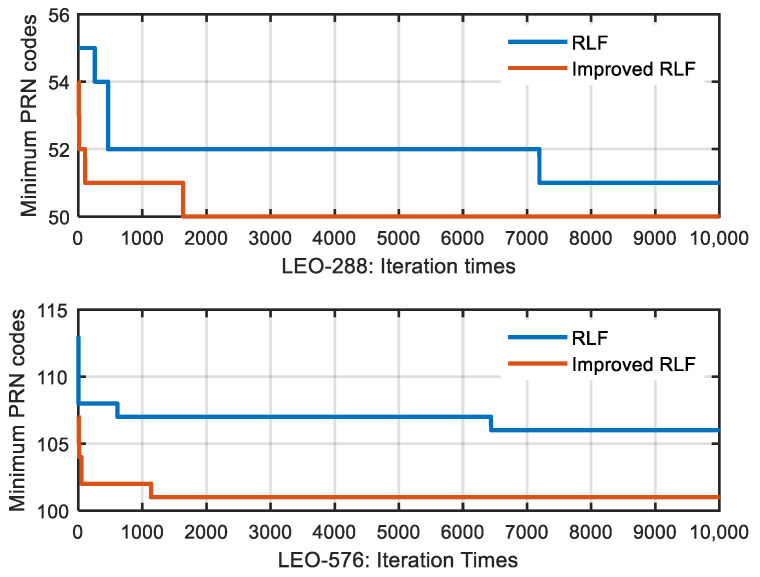
Comparison of minimum PRN codes between the RLF algorithm and the improved-RLF algorithm. The satellite configuration was the walker 288/12/6 at an altitude of 1000 km and the walker 576/24/12 at an altitude of 900 km. Both the RLF and improved-RLF were run once with 10,000 iterations.

From the experiment, we found that the improved RLF algorithm can get a smaller approximate optimal solution within a certain time period. As can be seen from Figure 6, with a probability of 0.0119, the approximate optimal solution of RLF algorithm was less than that of the improved-RLF with 6000 iterations. As the number of satellites increased, this happened more and more. Figure 8 shows how the minimum PRN codes of the RLF algorithm and improved-RLF algorithm changed with the number of iterations. We found that, compared with the improved RLF algorithm, the RLF algorithm required more iterations to obtain an approximate optimal solution and, in some cases, did not obtain it. Thus, the improved RLF algorithm can obtain a smaller approximate optimal solution within a certain period of time, and it is more suitable for calculating the minimum number of PRN codes. In the following section, we describe the use of the improved RLF algorithm to conduct experiments on satellite PRN code assignment.

## 4. Results

In this section, in order to study the effect of satellite orbital parameters on the number of minimum PRN codes, we take the polar-orbiting, walker, and hybrid constellations as experimental objects and come to the conclusions.

### 4.1. Polar-Orbiting Constellation

**Figure 9:**
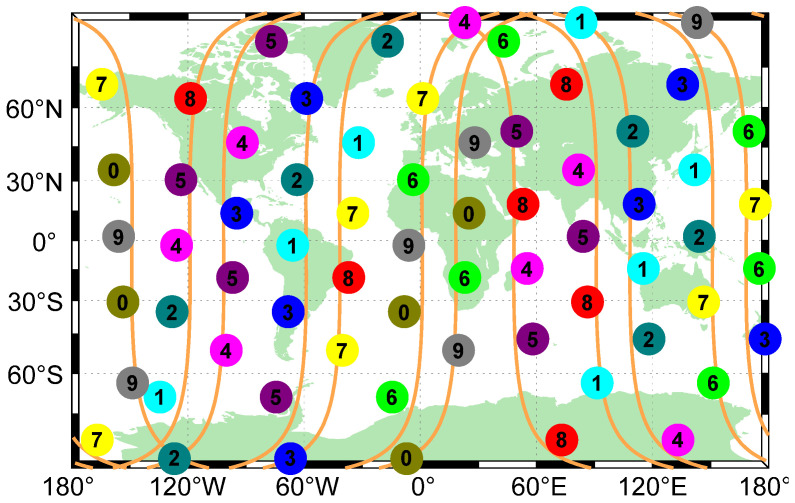
Graph of Iridium PRN code assignment. Ten colors and numbers represent ten PRN codes, which are enough to realize CDMA for Iridium constellation.

Since the navigation service provided by the GNSS has limitations for users in high-latitude regions, a polar-orbiting configuration has been widely used in LEO augmentation navigation systems due to the advantage of polar-orbiting constellations of traveling north-south over both poles of the Earth and covering different sections of the Earth’s surface. In this section, we take Iridium as an example and conduct the survey. Iridium consists of 66 satellites in 6 planes inclined at 52 degrees, and the orbital altitude is 778 km [38]. The configuration can be seen in Figure 4b. The result of the satellite PRN codes assignment is shown in Figure 9, and satellites broadcasting the same PRN code are shown to be far enough away from each other. These 10 PRN codes are sufficient for realizing CDMA and ranging of the Iridium constellation.

**Figure 10:**
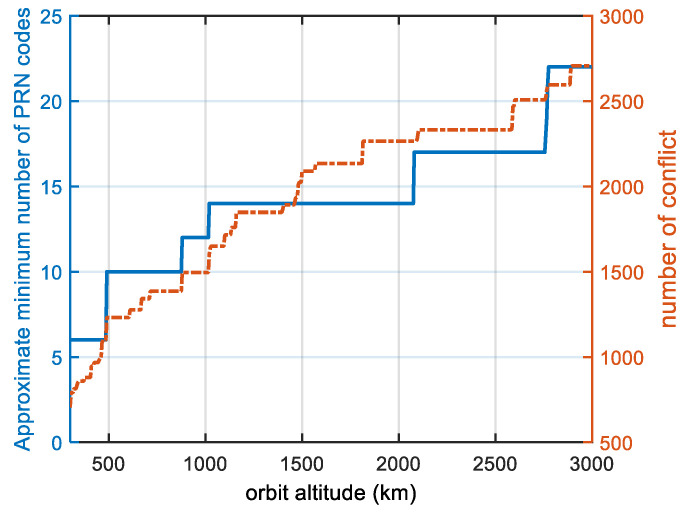
Graph of the orbital altitude and approximate minimum number of PRN codes. The blue line shows the approximate minimum number of PRN codes, and the orange line shows the number of conflicts. This is a polar-orbiting constellation at low altitudes of 300–3000 km, which is divided into 6 orbits with 11 satellites each. The number of conflicts is equal to the number of instances of 1 in the conflict matrix R.

In order to study the effect of the satellite orbital altitude on the number of minimum PRN codes, we changed the altitude of the satellite while keeping the other satellite parameters constant. Figure 10 shows the minimum number of PRN codes and the number of conflicts (the number of 1 in the conflict matrix R) for each altitude. The minimum number of PRN codes was found to be associated with the orbital altitude, and for a given constellation configuration and orbital inclination, the higher the altitude is, the more PRN codes are needed. When our proposed PRN code assignment strategy is applied, even if the LEO orbital altitude grows to 3000 m, 22 PRN codes are sufficient, which reduces the acquisition time by two-thirds and reduces the receiver’s memory resources required by two-thirds compared to the method where a unique PRN code is assigned to each satellite.

**Figure 11:**
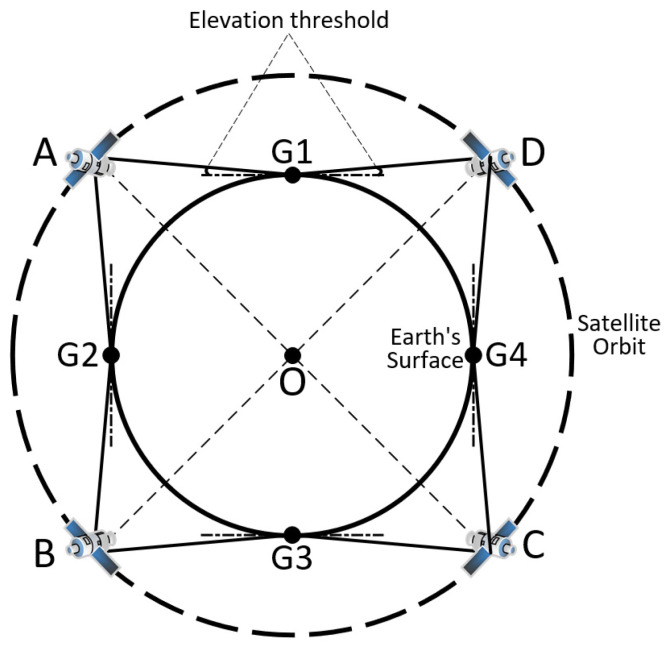
The critical state of two satellites observed by the same user. O is the center of the Earth, and G1, G2, G3, and G4 are the locations of users. A, B, C, and D are satellites with the same orbital altitudes, which are uniformly distributed.

As can be seen from Figure 10, the approximate minimum number of PRN codes and the number of conflicts increase in a step-by-step manner. This phenomenon can be explained by Figure 11, which shows the critical state. If the satellite orbital altitude becomes higher, users can receive signals from two satellites at the same time. If the satellite orbital altitude becomes lower, users cannot receive signals from two satellites at the same time. On the one hand, if the other orbital parameters remain unchanged, the higher the satellite orbit is, the greater the satellite signal coverage is, the more satellites that can be seen by users on the ground, and the greater the conflict relationship between satellites is, so more PRN codes are needed. On the other hand, as the satellite altitude increases, the number of conflicts is jumpy at the critical point in Figure 11 from 0 to 8, and these conflicts are A-B, B-A, B-C, C-B, C-D, D-C, D-A, and A-D. Because the distribution of satellites is uniform, the number of conflicts is jumpy at the critical point, and the approximate minimum number of PRN codes is jumpy, which is also characteristic of uniform constellations.

### 4.2. Walker Constellation

**Figure 12:**
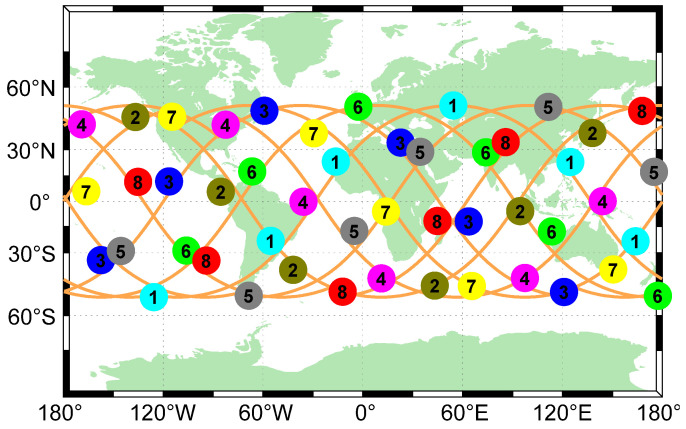
Graph of Globalstar grouping multiplexed PRN codes. The Globalstar constellation is a walker 52°:48/8/1 constellation with an altitude of 1414 km. Eight colors and numbers represent eight PRN codes, which are enough to realize CDMA for Globalstar constellation.

The walker configuration, which comes from [41], has been widely used in LEO augmentation navigation systems due to its symmetrical distribution and favorable global coverage. In this section, we take Globalstar as an example and conduct the survey. The Globalstar constellation is a walker 52°:48/8/1 constellation [21]. This means that there are 48 satellites in 8 planes inclined at 52 degrees, evenly spanning the 360° around the equator. The value “1” defines the phasing between the planes. The configuration can be seen in Figure 4a. The result of satellite PRN code assignment is shown in Figure 12, and these 8 PRN codes are shown to be sufficient for CDMA and ranging of the Globalstar constellation.

**Figure 13:**
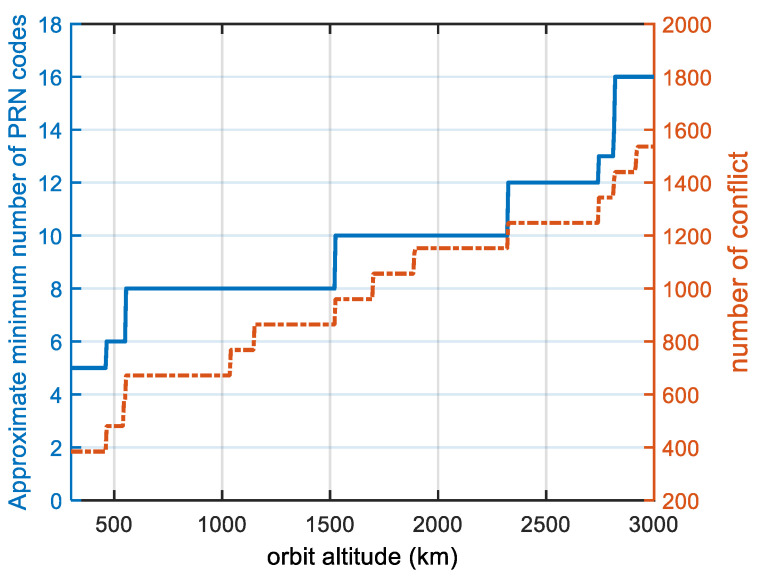
Graph of the orbital altitude and approximate minimum number of groups. The blue line shows the approximate minimum number of PRN codes, and the orange line shows the number of conflicts. This is a walker constellation similar to Globalstar, which is divided into 8 orbits with 11 satellites each. The orbital inclination is 52 degrees. The number of conflicts is equal to the number of instances of 1 in the conflict matrix R.

**Figure 14:**
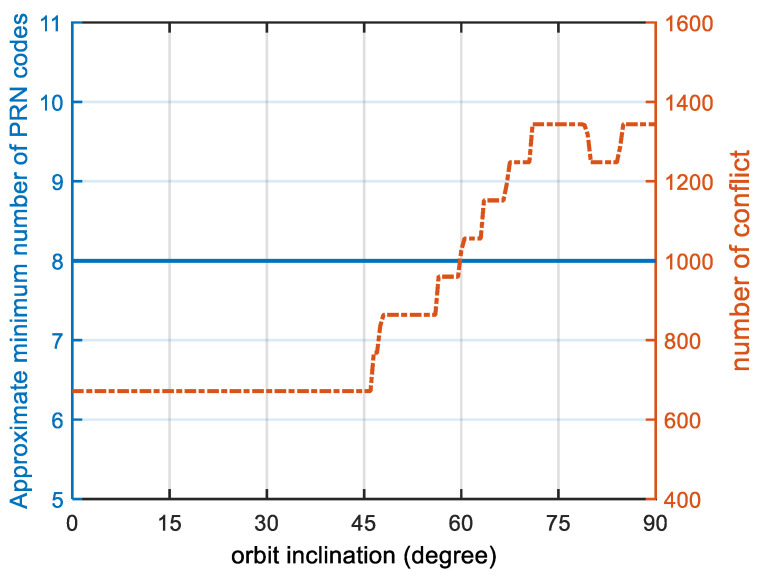
Graph of the orbital inclination and approximate minimum number of groups. The blue line shows the approximate minimum number of PRN codes, and the orange line shows the number of conflicts. This is a walker constellation similar to Globalstar, which is divided into 8 orbits with 11 satellites each. The orbital altitude is 1414 km. The number of conflicts is equal to the number of instances of 1 in the conflict matrix R.

We studied the effect of the satellite orbital altitude and inclination of the walker constellation on the number of the minimum PRN codes. Figure 13 and Figure 14 show the results for each altitude and inclination. On one hand, once again, we proved the conclusion that if other satellite parameters remain the same, the higher the altitude is, the more PRN codes needed. On the other hand, if the constellation configuration and satellite altitude remain the same, the minimum number of PRN codes is independent of the orbital inclination: no matter what the orbital inclination is, the minimum number of PRN codes remains the same.

### 4.3. Hybrid Constellation

We assumed that a hybrid constellation with N+M satellites can be divided into two constellations, each of which is a polar-orbiting or walker constellation, with different periods. The conflict matrix of the first constellation $\left\{v_{1}^{1},v_{2}^{1},\ldots ,v_{N}^{1}\right\}$ with N satellites is $R_{N\times N}^{1}$, while the conflict matrix of the other constellation $\left\{v_{1}^{2},v_{2}^{2},\ldots ,v_{M}^{2}\right\}$ with M satellites is $R_{M\times M}^{2}$. Through many experiments, we found that if there is no special design for the orbital period or orbital altitude, as time increases, the conflict matrix R tends to

(14) $$R_{N+M,N+M}=\left[\begin{array}{c} R_{N\times N}^{1}\;\;\;\;1_{N\times M}\;\\ 1_{M\times N}\;\;\;\;\;R_{M\times M}^{2} \end{array}\right]$$

where 1 is a matrix with all elements equal to 1.

**Figure 15:**
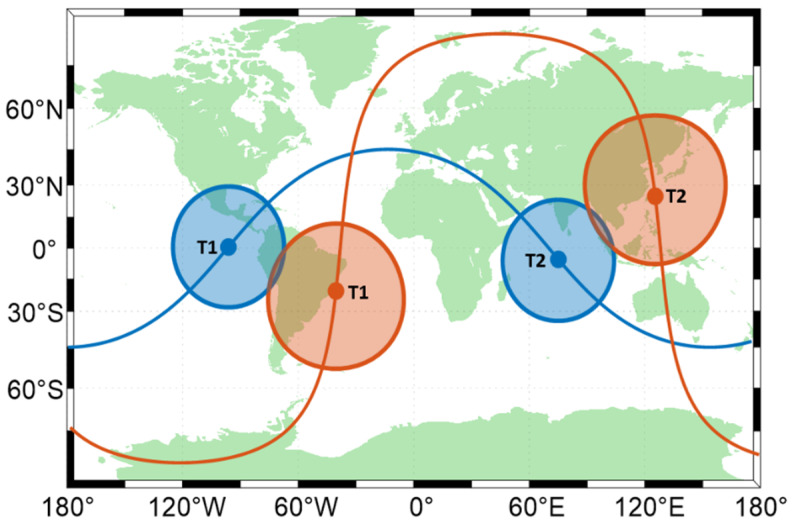
Diagram of sub-satellite points in different satellite orbits. These two different colors represent two satellites with different orbits. The points are satellite positions. The circles show the range in which the satellite PRN codes can be received on the ground.

This shows that any satellite in the first constellation conflicts with any satellite in the other constellation, so a hybrid constellation can form two independent sets. Therefore, the minimum PRN code number for a hybrid constellation is the sum of the two constellations. From Figure 15, it can also be seen that if the two satellites do not have a common period, the two satellite signals are likely to be observed by users near the ground where the orbital planes intersect, so the two satellite cannot share the same PRN code. In the same way, a constellation composed of multiple polar-orbiting or walker constellations also has the above characteristics.

In summary, for hybrid constellations, we recommend that, after calculating the minimum number of PRN codes for each polar-orbiting or walker constellation separately, the minimum PRN code number of the hybrid constellation should be equal to the sum of the minimum PRN codes in the partial constellations that make up the hybrid constellation.

## 5. Discussion

The traditional method of satellite PRN code assignment is suitable for GNSS constellations consisting of dozens of satellites. However, LEO constellations have huge satellite numbers compared with the GNSS. These constellations may operate as navigation systems and may broadcast navigation signals in the future, placing a huge burden on the navigation receiver. Therefore, a novel satellite PRN code assignment method needs to be proposed.

As aforementioned, the novel method in which several satellites share the same PRN code was shown to be feasible and beneficial for the navigation receiver and PRN code design. For most LEO constellations, the storage resources and the average satellite acquisition time of the receiver will be between one-fifth and one-tenth of the previous values when this method is used. Even if the orbital altitude increases to 3000 km, the storage resources and the average satellite acquisition time of the receiver will be about one-third of the previous values. This method can also greatly reduce the design difficulty of PRN codes.

For hybrid constellations, we suggest that the minimum number of PRN codes is calculated for each polar-orbiting or walker constellation separately at first. For constellations proposed by different companies, we also suggest that the minimum number of PRN codes is calculated for each constellation separately. This can avoid the mixing of signals from different satellites sharing the same PRN code as much as possible.

In terms of algorithms, we believe that several algorithms can be used for obtaining the satellite PRN code assignment scheme, but in order to minimize the number of PRN codes as much as possible, the RLF algorithm was selected and improved. If there are new algorithms available for graph coloring in the future, we will try to apply them to the satellite PRN code assignment problem.

It should be pointed out that the conclusions of this paper were obtained under the condition that the constellation configuration remains unchanged. However, the actual satellite is affected by various perturbation forces, and the orbital parameters of the satellite will shift and change periodically. Therefore, in future work, after making the satellite orbit more precise, it will be possible to obtain better satellite PRN code assignment results.

## 6. Conclusions

Facing hundreds of LEO navigation enhancement satellites in the future, in order to shorten the storage resources and acquisition time of the navigation receiver, we put forward the idea of several LEO satellites sharing the same PRN code. We investigated the benefits of this novel method compared with the current method of assigning a unique PRN code to each satellite. The conclusions are summarized as follows:

1. It is feasible and meaningful for several satellites sharing the same PRN code to save storage resources and reduce the satellite acquisition time of the receiver.
2. The RLF algorithm is effective for calculating the minimum number of PRN codes, and the improved-RLF algorithm accelerates the calculation speed.
3. In terms of satellite orbital parameters of polar-orbiting or walker constellations, the higher the altitude is, the more PRN codes needed. If other satellite parameters remain the same, no matter what the orbital inclination is, the minimum number of PRN codes remains the same.
4. In terms of hybrid constellations, we recommend that, after calculating the minimum number of PRN codes for each polar-orbiting or walker constellation separately, the minimum PRN code number of a hybrid constellation will be equal to the sum of the minimum PRN code numbers of the partial constellations that make up the hybrid constellation.

Overall, if this new technology is applied, the storage resources of the receiver will be, at most, one-third of the previous value, and likewise, the average satellite acquisition time will be, at most, one-third of what it used to be. This new method can also greatly reduce the design difficulty of PRN code.

## Figures and Tables

**Table 1:** Summary of some commercial LEO constellations.

Constellation	Country	No. Satellites	Altitude (km)	Inclination (deg)	Year
Iridium [10]	USA	66	781	86.4	1998
Globalstar [21]	USA	48	1400	52	2000
Iridium NEXT [22]	USA	75	780	86.4	2019
LeoSat [23]	USA	108	1400	-	2019/2020
SpaceX Starlink [17]	USA	1600	1110	53.8	2024
		400	1130	74	
		1600	1150	53	
		375	1275	81	
		450	1325	70	
		7518	340	-	-
Boeing [18]	USA	1190	1200	45	2027
		612		55	
		1155		88	
OneWeb [16]	USA/UK	648	1200	88	2027
Hongyun [24]	China	156	1000		2022
Hongyan [19]	China	54	1100	-	2023
		324	-	-	-
Telesat [25]	Canada	72	1000	99.5	2021
		45	1248	37.4	
Astrome Technology [26]	India	150	1400	-	-
Samsung [27]	Korea	4600	1400	-	-

**Table 2:** Detailed orbital configurations and approximate minimum solution of the designed LEO constellations.

Constellation Name	Type of Orbit	Number	Altitude (km)	Inclination (deg)	Planes Number	Approximate Minimum	Ratio
LEO-48	Walker	48	1414	52	8	8	6
LEO-66	Polar	66	778	86.4	6	10	6.6
LEO-120	Walker	120	1000	55	12	16	7.5
LEO-192	Polar	192	1200	90	12	34	5.65
LEO-288	Polar	288	1000	98.2	12	50	5.76
LEO-576	Walker	576	900	60	24	101	5.70

## Data Availability

Not applicable.
